# Investigation to analyse the correlation between ‘human plumbline distance’, ‘formetric plumbline distance’ and ‘inclinometry’ in a clinical setting: a pilot correlational study

**DOI:** 10.1186/1748-7161-9-S1-O4

**Published:** 2014-12-04

**Authors:** Jason Black, Erika Maude, Juliet Mayes, David Glynn, Michael Bradley

**Affiliations:** 1 Scoliosis SOS Clinic, London, UK; 2 Independent statistician, London, UK

## Background

Sagittal plane measures are an important aspect of evaluation of patients with both Adolescent Idiopathic Scoliosis (AIS) and Hyperkyphosis. It is currently advised that the sagittal profile of AIS patients is measured clinically using Plumbline Distance (PD), yet competence of its use in clinical practice has not been regularly evaluated [3].

## Aims

To determine the correlation between Human PD (HPD), Formetric PD (FPD) and Inclinometry measured clinically.

## Design

Pilot correlational study.

## Methods

27 consecutive female AIS patients aged 11-18 were measured prior to commencement of ScolioGold physiotherapy treatment with HPD, FPD and Inclinometry with measures repeated on completion of treatment. All measures were taken by one therapist (JM). FPD data considered was Cervical Apex (CA), Vertebral Prominence (VP), Lordotic Apex (LA) and Dimple Middle (DM).

Correlation was studied between singular measures of HPD vs. FPD, between predicted Kyphosis (C7+L3 vs VP+LA) and between both HPD and FPD vs. Inclinometry (C7+L3 or VP+LA vs Angle A+B).

## Results

An initial analysis for normality (‘Shapiro-Wilk’ and ‘Skew and Kurtosis’) showed normal distributions in all variables except VP as measured by FPD and S1 as measured by HPD. Thus Pearson’s correlation was used in all cases except C7 vs. VP and S1 vs DM which utilised Spearman’s Correlation.

A strong positive correlation was shown between measures of HPD and FPD (Cervical vs. CA [r=0.574], C7 vs. VP [r=0.542], L3 vs. LA [r=0.683], S1 vs. DM [r=0.570]). A very strong correlation was demonstrated between kyphosis predictors in HPD and FPD (C7+L3 vs. VP+LA [r=0.782]). Similarly, Inclinometry showed a strong correlation with kyphosis predictors in both HPD and FPD (A+B vs. C7+L3 [r=0.563] or VP+LA [r=628]).

Investigative analysis of data demonstrated pre-treatment Inclinometer Kyphosis (A+B) as 24 degrees (SD 7.96) and FPD (VP+LA) as 50.77mm (SD 19.04).

## Conclusion

This study has justified the use of HPD or Inclinometry in the absence of FPD in clinical settings to evaluate sagittal profiles in female patients with AIS for this small patient group. Future studies should be done to evaluate clinical correlation between radiographic kyphosis and HPD or Inclinometry, as well as considering the potential effect of inter-rater error.
